# Mutant p53 uses p63 as a molecular chaperone to alter gene expression and induce a pro-invasive secretome

**DOI:** 10.18632/oncotarget.382

**Published:** 2011-12-25

**Authors:** Paul M. Neilsen, Jacqueline E. Noll, Rachel J. Suetani, Renee B. Schulz, Fares Al-Ejeh, Andreas Evdokiou, David P. Lane, David F. Callen

**Affiliations:** ^1^ Cancer Therapeutics Laboratory, Discipline of Medicine, University of Adelaide, Australia; ^2^ Signal Transduction Laboratory, Queensland Institute for Medical Research, Australia; ^3^ Discipline of Surgery, Basil Hetzel Institute, University of Adelaide, Australia; ^4^ p53Lab, Immunos, Agency for Science, Technology and Research, Singapore

**Keywords:** mutant p53, p63, secretome, invasion

## Abstract

Mutations in the *TP53* gene commonly result in the expression of a full-length protein that drives cancer cell invasion and metastasis. Herein, we have deciphered the global landscape of transcriptional regulation by mutant p53 through the application of a panel of isogenic H1299 derivatives with inducible expression of several common cancer-associated p53 mutants. We found that the ability of mutant p53 to alter the transcriptional profile of cancer cells is remarkably conserved across different p53 mutants. The mutant p53 transcriptional landscape was nested within a small subset of wild-type p53 responsive genes, suggesting that the oncogenic properties of mutant p53 are conferred by retaining its ability to regulate a defined set of p53 target genes. These mutant p53 target genes were shown to converge upon a p63 signalling axis. Both mutant p53 and wild-type p63 were co-recruited to the promoters of these target genes, thus providing a molecular basis for their selective regulation by mutant p53. We demonstrate that mutant p53 manipulates the gene expression pattern of cancer cells to facilitate invasion through the release of a pro-invasive secretome into the tumor microenvironment. Collectively, this study provides mechanistic insight into the complex nature of transcriptional regulation by mutant p53 and implicates a role for tumor-derived p53 mutations in the manipulation of the cancer cell secretome.

## INTRODUCTION

The p53 tumor suppressor plays a critical role in the prevention of oncogenic transformation through the elimination or permanent growth arrest of potentially malignant cells. Upon cellular insults, p53 is activated and functions as a sequence-specific transcription factor to regulate the expression of specific genes, thereby inducing DNA repair, cell-cycle arrest, apoptosis, and senescence [1, 2]. However, approximately 50% of all human cancers harbour mutations in the *TP53* gene, commonly resulting in expression of a full-length protein with a single amino acid substitution [3]. These tumors typically have mutations at specific residues (R175, G245, R248, R249, R273 and R282) within their DNA-binding domain, and express high levels of the mutated p53 proteins [4]. In contrast to the tumor suppressive effects of wild-type p53, mutant p53 proteins have been shown to promote cancer progression by enhancing the ability of cancer cells to invade and metastasize [5-10], confer resistance to chemotherapies [11, 12], promote genomic instability [13, 14] and drive multinucleation [15]. These observations strongly indicate that mutant p53 possesses gain-of-function properties that promote oncogenesis.

A diverse array of molecular mechanisms have been proposed to explain the oncogenic influence of mutant p53 during cancer development and progression. A widely accepted gain-of-function mechanism is the ability of mutant p53 to both physically interact with and inactivate the p53 family member, p63 [5, 6]. p63 is a transcription factor that plays a pivotal role in development and can be expressed as two isoforms that either have an intact (TAp63) or deleted (ΔNp63) amino-terminal transactivation domain [16]. The full length isoform, TAp63, has genuine tumor suppressor traits as it can activate genes to inhibit metastasis and promote apoptosis or cell cycle arrest [17, 18]. Mutant p53 has been shown to bind and sequester TAp63 away from its target genes, thereby hampering its anti-metastatic capacity [5, 16, 19]. Our current understanding of this complex relationship between mutant p53 and p63 is restricted to this antagonistic model.

In this study, we discover an unprecedented role of p63 in the gene regulation network of mutant p53 through global gene profiling analyses. For the first time, we show that mutant p53 uses p63 as a molecular chaperone to tether to the promoters of its target genes. Through p63, mutant p53 aberrantly alters the gene expression pattern of cancer cells to promote oncogenesis. In addition, we also reveal the capability of mutant p53 to manipulate the secretome of cancer cells as a novel mechanism to drive invasion.

## RESULTS

### Inducible cell lines as a tool to study the oncogenic functions of mutant p53

**Figure 1:**
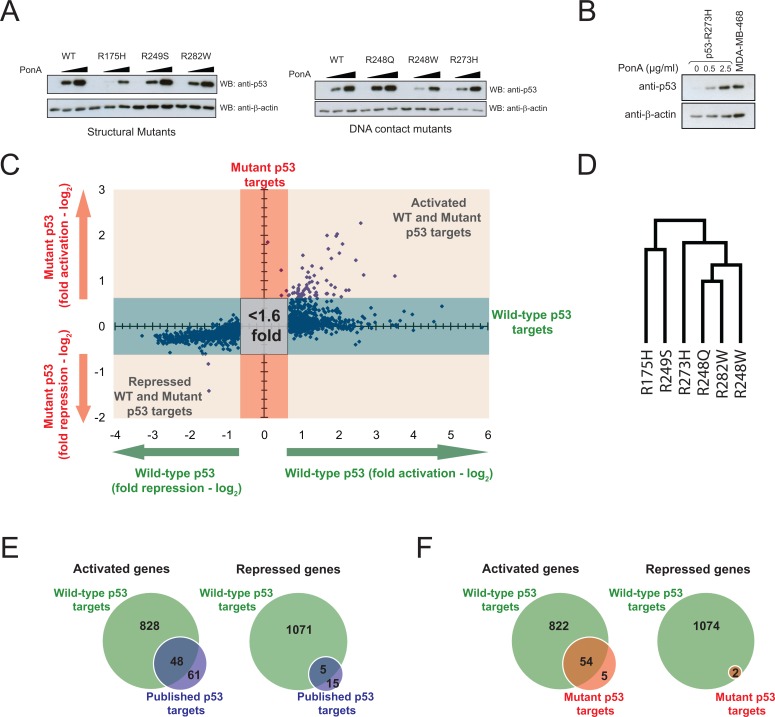
Expression microarray analysis of inducible wild-type and mutant p53 in H1299 cells (A) The EI-H1299 cell lines expressing wild-type or mutant p53 show inducible expression of p53 protein following 24 hours induction with 0, 0.5 or 2.5 μg/mL of the inducing agent (PonA). (B) EI-H1299 cells with inducible p53 R273H were cultured with the indicated concentration of PonA. Western blot analysis was used to determine the total p53 R273H levels in comparison with endogenous p53 R273H expressed in the MDA-MB-468 breast cancer cell line. β-actin was used as a loading control. (C) Scatterplot of expression array data including genes regulated by >1.6 fold by either wild-type p53 or across all six p53 mutants. (D) Hierarchical clustering of transcriptional regulation by each p53 mutant, as determined using Gene Pattern 2.0 [45]. (E) Venn diagram illustrating the overlap between genes regulated by wild-type p53 in this expression microarray analysis as compared with known *bone fide* direct p53 target genes [20]. (F) Venn diagram illustrating the overlap between genes regulated by mutant p53 (Table 1) and wild-type p53 in this expression microarray analysis.

The study of the precise function of mutant p53 in cancer is generally hampered by the broad spectrum of different *TP53* mutations and the diverse genetic backgrounds of mutant p53-expressing cancer cell lines. To overcome these challenges, we have used the H1299 cell line with a p53 null background for the inducible expression of six common p53 hot spot mutants (R175H, R248Q, R248W, R249S, R273H and R282W) and the wild-type p53 as a control (Fig. 1A). Initial phenotypic analysis of these inducible p53 cell lines showed that the induction of wild-type p53 resulted in a growth arrest at the G_1_ phase of the cell cycle, while induction of the p53 mutants did not influence proliferation (Fig. S1). Our previously published data demonstrated that inducible expression of p53 mutants, but not the wild-type counterpart, endowed the cells with oncogenic properties, including the ability to drive invasion, epithelial-to-mesenchymal transition (EMT) and centrosomal abnormalities [15]. Importantly, the relative levels of induced mutant p53 expression were comparable to the levels of endogenous p53 R273H observed in the MDA-MB-468 breast cancer cell line, suggesting that the inducible system produces physiologically relevant amounts of mutant p53 (Fig. 1B). Collectively, these results indicated that the inducible mutant p53 cell lines generated in this study are highly physiologically-relevant and can be used as a sensitive expression platform to capture the oncogenic events during transcriptional reprogramming by mutant p53.

**Table 1:** Genes regulated by wild-type or mutant p53

	Accession Number	Gene Symbol	Gene Name	Fold Change	
				Mut p53	WT p53
**Wild-type and Mutant p53 Activated**	NM_002560	**P2RX4**	Purinergic receptor P2X, ligand-gated ion channel, 4	4.81	6.02
	NM_006528	**TFPI2**	Tissue factor pathway inhibitor 2	4.08	3.88
	NM_001002236	**SERPINA1**	Serpin peptidase inhibitor, clade A	3.99	3.21
	NM_002905	**RDH5**	Retinol dehydrogenase 5	3.95	3.81
	NM_025181	**SLC35F5**	Solute carrier family 35, member F5	3.50	4.28
	NM_199511	**CCDC80**	Coiled-coil domain containing 80	3.25	3.29
	NM_001902	**CTH**	Cystathionase	3.05	4.04
	NM_003764	**STX11**	Syntaxin 11	2.95	2.05
	NM_002599	**PDE2A**	Phosphodiesterase 2A	2.81	3.48
	NM_012242	**DKK1**	Dickkopf homolog 1	2.74	5.49
	NM_000358	**TGFBI**	Transforming growth factor, beta-induced	2.51	2.43
	BC071561	**LRIG1**	Leucine-rich repeats and immunoglobulin-like domains 1	2.43	2.03
	NM_032169	**ACAD11**	Acyl-Coenzyme A dehydrogenase family, member 11	2.42	2.90
	NM_021947	**SRR**	Serine racemase	2.28	2.26
	NM_019058	**DDIT4**	DNA-damage-inducible transcript 4	2.19	6.35
	NM_000332	**ATXN1**	Ataxin 1	2.17	2.53
	NM_006622	**PLK2**	Polo-like kinase 2	2.15	11.34
	BC025968	**BHLHB3**	Basic helix-loop-helix domain containing, class B, 3	2.12	2.21
	NM_020946	**DENND1A**	DENN/MADD domain containing 1A	2.10	2.33
	NM_001957	**EDNRA**	Endothelin receptor type A	2.01	3.19
	NM_002756	**MAP2K3**	Mitogen-activated protein kinase kinase 3	1.98	2.43
	NM_015310	**PSD3**	Pleckstrin and Sec7 domain containing 3	1.95	2.32
	NM_003012	**SFRP1**	Secreted frizzled-related protein 1	1.94	2.69
	NM_021021	**SNTB1**	Syntrophin, beta 1	1.88	2.20
	NM_005860	**FSTL3**	Follistatin-like 3	1.88	3.46
	NM_006738	**AKAP13**	A kinase (PRKA) anchor protein 13	1.88	1.91
	NM_005562	**LAMC2**	Laminin, gamma 2	1.82	2.20
	NM_003155	**STC1**	Stanniocalcin 1	1.81	1.72
	NM_001966	**EHHADH**	Enoyl-Coenzyme A, hydratase/3-hydroxyacyl Coenzyme A dehydrogenase	1.81	3.73
	NM_015046	**SETX**	Senataxin	1.79	1.88
	BC004121	**OCEL1**	Occludin/ELL domain containing 1	1.77	2.13
	AY358949	**TMEM205**	Transmembrane protein 205	1.75	1.88
	NM_033446	**FAM125B**	Family with sequence similarity 125, member B	1.74	4.83
	NM_006762	**LAPTM5**	Lysosomal associated multispanning membrane protein 5	1.73	4.57
	NM_004780	**TCEAL1**	Transcription elongation factor A (SII)-like 1	1.72	2.08
	NM_005100	**AKAP12**	A kinase (PRKA) anchor protein (gravin) 12	1.72	1.88
	NM_139314	**ANGPTL4**	Angiopoietin-like 4	1.68	2.62
	NM_015990	**KLHL5**	Kelch-like 5	1.67	1.75
	NM_003619	**PRSS12**	Protease, serine, 12	1.66	1.84
	NM_003326	**TNFSF4**	Tumor necrosis factor (ligand) superfamily, member 4	1.66	2.03
	NM_006621	**AHCYL1**	S-adenosylhomocysteine hydrolase-like 1	1.66	1.70
	NM_005347	**HSPA5**	Heat shock 70kDa protein 5	1.65	2.02
	NM_006226	**PLCL1**	Phospholipase C-like 1	1.65	3.23
	NM_153268	**PLCXD2**	Phosphatidylinositol-specific phospholipase C, X domain containing 2	1.64	3.18
	NM_021623	**PLEKHA2**	Pleckstrin homology domain containing, family A	1.63	1.94
	NM_138578	**BCL2L1**	BCL2-like 1	1.62	1.89
	NM_006379	**SEMA3C**	Sema domain, immunoglobulin domain (Ig), short basic domain, secreted 3C	1.62	3.65
	NM_016303	**WBP5**	WW domain binding protein 5	1.62	2.09
	NM_000960	**PTGIR**	Prostaglandin I2 (prostacyclin) receptor	1.61	2.00
	NM_001083899	**GP6**	Glycoprotein VI	1.61	2.65
	NM_001080503	**CCDC159**	Coiled-coil domain containing 159	1.61	2.57
	NM_002204	**ITGA3**	Integrin, alpha 3	1.60	2.62
**WT and Mut p53 Repressed**	NM_001801	**CD22**	CD22 molecule	−2.67	−2.80
	NM_001771	**CDO1**	Cysteine dioxygenase, type I	−1.77	−2.83
**Mutant p53 Activated**	NM_152637	**METTL7B**	Methyltransferase like 7B	3.60	
	NM_005291	**GPR17**	G protein-coupled receptor 17	2.35	
	NM_020698	**TMCC3**	Transmembrane and coiled-coil domain family 3	1.72	
	NM_021005	**NR2F2**	Nuclear receptor subfamily 2, group F, member 2	1.60	
	NM_001098817	**INO80C**	INO80 complex subunit C	1.60	

### Deciphering the global landscape of transcriptional regulation by mutant p53

In order to decipher the global gene regulation network of mutant p53, expression microarrays were performed on the inducible mutant p53 cell lines after 24 hours of induction (Fig. 1C). The gene expression profiles for each p53 mutant were determined using paired induced and un-induced cultures, thus providing a sensitive assessment of genes specifically expressed in the presence of the induced mutant p53. Gene profiling analysis revealed that the ability of mutant p53 to alter the transcriptional profile of cancer cells is remarkably conserved across different mutants, as R175H, R248Q, R248W, R249S, R273H and R282W all regulated a core set of 59 genes (Table 1). Surprisingly, the hierarchical clustering of the expression profiles for the hot spot p53 mutants studied did not correlate with their previously attributed ‘DNA contact’ or ‘structural’ properties. In fact, there was no relationship between the tertiary structure of the p53 mutant and its transcriptional regulation (Fig. 1D).

**Figure 2:**
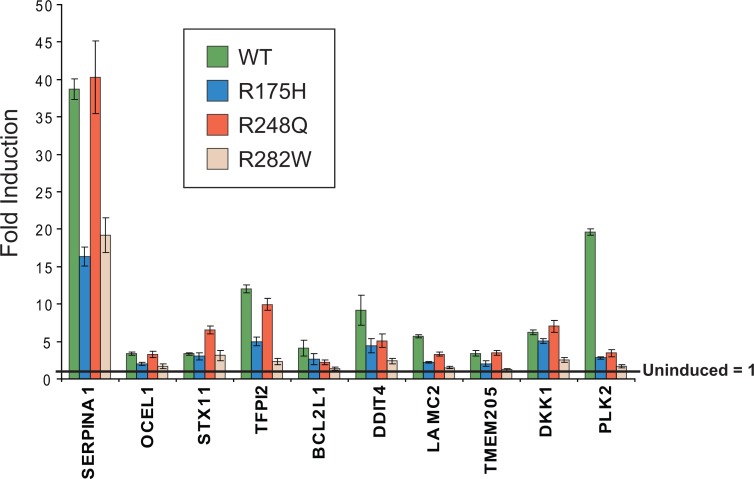
Validation of target genes identified in EMA Ten target genes identified as regulated by >1.6-fold in the inducible p53 mutant cell lines were validated in the inducible cell lines. EI-H1299 p53-WT, R175H, R248Q or R282W cell lines were cultured in the presence of PonA (2.5 μg/mL) or vehicle control for 24 hours and the expression of genes determined by specific real-time RT PCR analysis. Fold induction of target genes is presented relative to the uninduced control for each cell line (uninduced = 1).

To ascertain if the regulation of the core set of 59 genes is a unique property of mutant p53, expression microarray analysis of the wild-type p53 inducible cell line was performed in parallel. It was found that 1952 genes were regulated by wild-type p53 in this system and there was a considerable overlap between these genes with the published *bone fide* targets of wild-type p53 [20] (Fig. 1E). Interestingly, the majority of the core genes (54/59) identified from the gene profiling of p53 mutants were also modulated by wild-type p53, although this represents only 3% of the total wild-type targets (54/1952) (Fig. 1F). To validate our observations from the expression microarrays, we subsequently determined the expression of ten putative targets of wild-type and mutant p53 through quantitative real-time PCR analysis (Fig. 2). Indeed, all ten genes were significantly upregulated upon induction of either wild-type p53 or the p53 R175H, R248Q or R282W mutants, albeit to differing extents. Importantly, the inducing agent (PonA) did not up-regulate the expression of these genes in the parental (p53 deficient) H1299 cell inducible line (Fig. S2). In order to prove that the H1299 inducible system utilized in this study was indeed a genuine representation of the wild-type p53 response, we showed that inducible expression of wild-type p53, but not the mutant form, was able to transactivate the classical “tumor suppressor” targets including *p21*, *FAS*, *GADD45A* and *MDM2* (Fig. S3). Thus, we concluded that these core targets transactivated by both wild-type and mutant p53 may represent a set of genes that are functionally distinct from the majority of tumour suppressor target genes transactivated by wild-type p53.

**Figure 3:**
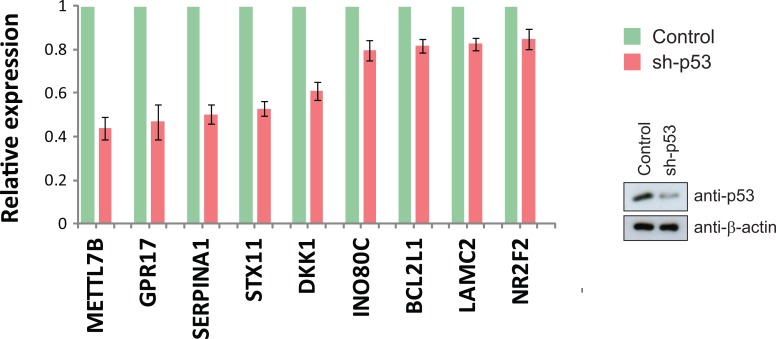
Endogenous mutant p53 regulates gene expression Silencing of endogenous mutant p53 R273H expression in MDA-MB-468 cells by a specific short hairpin RNA (sh-p53) resulted in a decrease in the basal expression of the indicated mutant p53 target genes.

Our global gene expression profiling of mutant p53 in H1299 cells revealed five genes, *METTL7B, GPR17, TMCC3, NR2F2* and *INO80C*, that are specifically up-regulated by all p53 mutants, but not wild-type p53 (Table 1). We investigated if these genes (plus the previously validated mutant p53 target genes from Fig. 2) were also regulated by endogenous mutant p53. Knockdown of endogenous p53 R273H in MDA-MB-468 cells resulted in a reduction of expression of METTL7B, GPR17, SERPINA1, STX11, DKK1, INO80C, BCL2L1, LAMC2 and NR2F2, thus implicating these genes as constitutively activated targets of an endogenously-expressed mutant p53 (Fig. 3).

**Figure 4:**
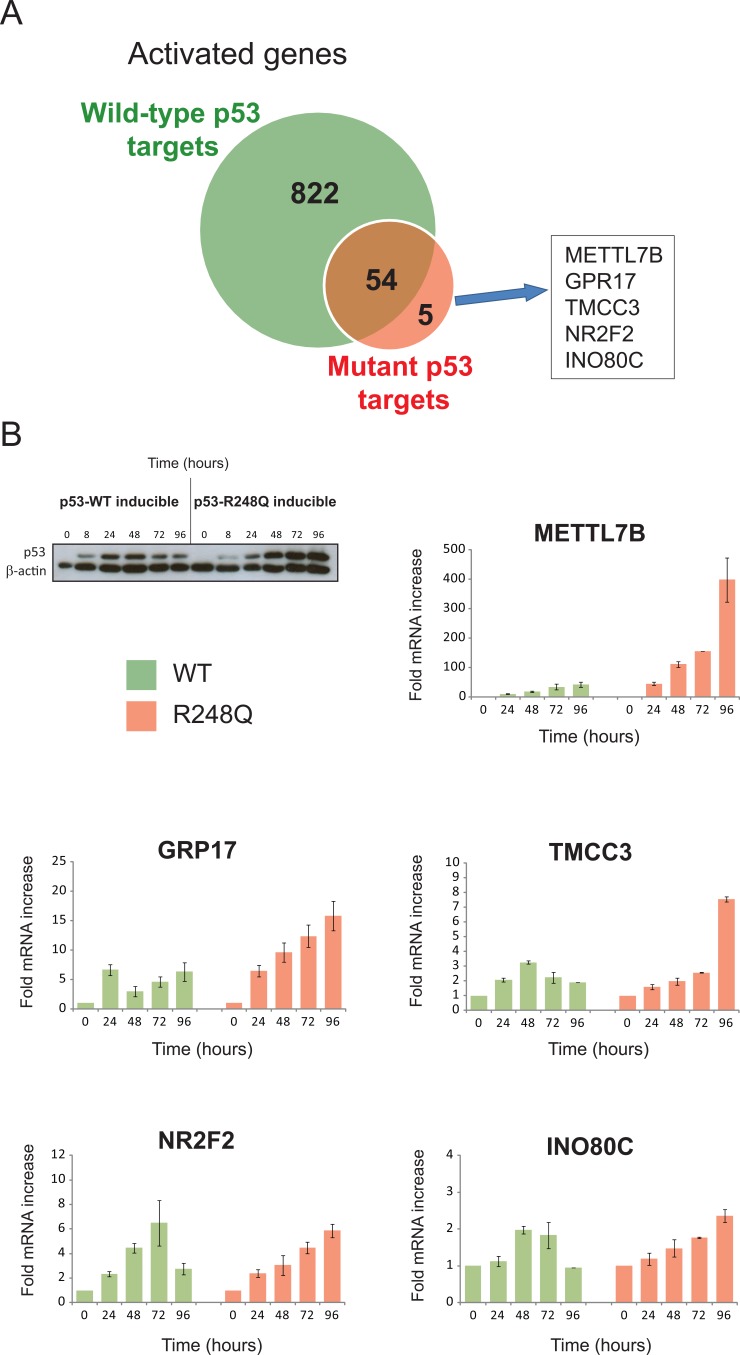
“Mutant specific” target genes are also wild-type p53 targets with altered induction kinetics (A) Five genes were identified from the expression microarray analysis as specifically up-regulated (>1.6-fold) in the mutants but not the WT inducible cell lines. (B) EI-H1299 cells with either inducible wild-type p53 or the p53 R248Q mutant were cultured with PonA (2.5 μg/mL) to induce p53 protein expression for 0, 24, 48, 72 and 96 hours and the expression of METTL7B, GPR17, TMCC3, NR2F2 and INO80C were determined by specific real-time RT PCR analysis. It is noteworthy that the induction of wild-type p53, but not the p53 R248Q mutant over this timecourse was associated with altered growth kinetics (see Supplementary Figure S1A).

We next assessed the kinetics of target gene transactivation by mutant p53 over an extended time course using real-time PCR. The five mutant p53-specific targets were selected for this study (Fig 4A). Transactivation kinetics were also examined following wild-type p53 induction to ensure that these genes were genuine mutant p53-specific targets. Surprisingly, wild-type p53 could also significantly increase the expression of all of these genes, albeit to a lesser extent and with altered kinetics (Fig. 4B). Collectively, these studies have revealed that the mutant p53 transcriptional landscape is nested within a small subset of wild-type p53 responsive genes. We propose that the shared wild-type and mutant p53 target genes identified through gene expression profiling represent the oncogenic transcriptional activities of p53. Indeed, none of these 59 genes are present in the list of *bone fide* p53 target genes responsible for its tumor suppression activities [21].

### Mutant p53 target genes involve the canonical p63 signalling network

**Figure 5:**
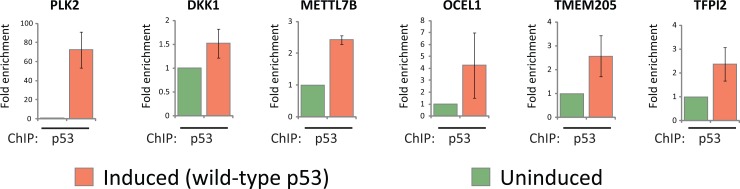
Wild-type p53 is associated with the promoters of mutant p53 target genes EI-H1299 with inducible expression of wild-type p53 were cultured in the presence of PonA (2.5 μg/mL) or vehicle control for 24 hours prior to ChIP analysis using a p53-specific antibody. The putative p53/p63-REs within the indicated gene promoters were located at the following positions from the initiation site (PLK2 ^−^2207bp; DKK1 [23]; METTL7B ^−^4993bp; OCEL1 ^−^6934bp; TMEM205 ^−^2538bp; TFPI2 ^−^7021bp).

We next explored if the altered expression levels of the 59 common targets of wild-type and mutant p53 were mediated through a direct or indirect mechanism. Intriguingly, the novel mutant p53 target genes identified from expression profiling included genes previously published as direct targets of p53 (*PLK2, DKK1* and *DDIT4*) [22-24]. We therefore employed an *in silico* approach to further explore a possible involvement of canonical p53 regulation of the 59 novel mutant p53 target genes listed in Table 1. Indeed, p53scan revealed that 54% (32/59) of these mutant p53 targets contained at least one putative p53 response element (RE) in their upstream promoter region, first intron or 3'UTR (Table S1A). To experimentally validate this *in silico* analysis, we selected six genes (*PLK2, DKK1, METTL7B, OCEL1, TMEM205* and *TFPI2*) for chromatin immunoprecipitation (ChIP) with wild-type p53. Indeed, ChIP analyses subsequently confirmed the recruitment of wild-type p53 to putative p53-REs identified in all six of these gene promoter regions (Fig. 5).

**Figure 6:**
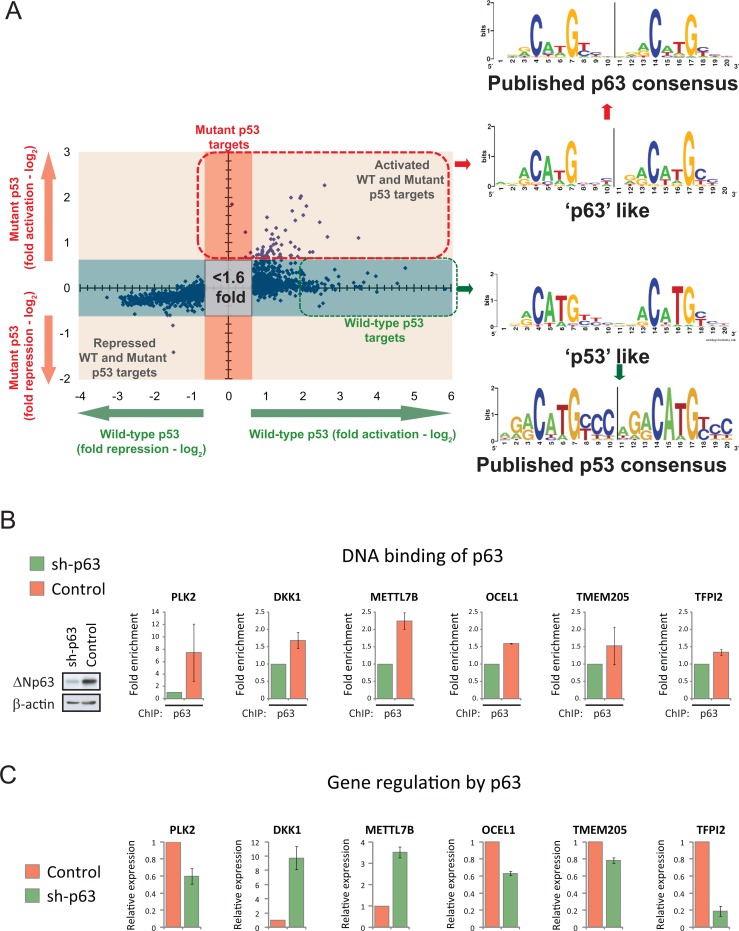
Mutant p53 regulated genes are direct targets of p63 (A) A p53 binding motif was derived from the putative p53-RE sequences identified in the promoter regions of mutant p53 target genes using p53scan software (red box; listed in Table S1A). A second p53 binding motif was derived from the putative p53-RE sequences identified in the 10kB upstream promoter regions of the indicated 59 wild-type p53-specific target genes using p53scan software (green box; listed in Table S1B). These p53 binding motifs were compared to the published consensus binding sequences for p53 [25] or p63 [26]. (B) Polyclonal populations of MCF10A cells were generated expressing either p63-specific shRNA (sh-p63) or non-targeting shRNA (control), with reduced p63 protein levels confirmed by western blot analysis. These cell lines were used for ChIP analysis to confirm the association of p63 with the indicated gene promoters at the following positions from the initiation site (PLK2 ^−^2207bp; DKK1 [23]; METTL7B ^−^4993bp; OCEL1 ^−^6934bp; TMEM205 ^−^2538bp; TFPI2 ^−^7021bp) (C) The relative expression of the indicated genes was determined in MCF-10A sh-p63 or control cells using real time PCR.

Interestingly, we observed that the p53 consensus binding sequence derived from the promoters of the mutant p53 target genes differs subtly from that of the published p53-RE [25] (Fig. 6A). Furthermore, this mutant p53-RE sequence also deviated subtly from the p53-REs identified in genes uniquely transactivated by wild-type p53 in our expression profiling (Fig. 6A; Table S1B). In fact, these identified p53 binding sites in the promoters of the mutant p53 target genes resembled more closely the published p63-RE [26] (Fig. 6A). Therefore, we speculated that these genes may also represent direct p63 target genes. We performed p63scan and identified a similar frequency of putative p63-REs in the regulatory elements of these 59 mutant p53 target genes (Table S1C). Next, we investigated if endogenous p63 could associate with the putative p63 binding sites in the six mutant p53 targets with validated p53-REs. ChIP analyses demonstrated that silencing of endogenous p63 significantly reduced the amount of p63 bound to the p63-REs in the promoter regions in all six genes tested (*PLK2, DKK1, METTL7B, OCEL1, TMEM205* and *TFPI2*) in the non-malignant MCF10A breast epithelial cell line (Fig. 6B). We also examined if p63 constitutively regulated the expression of these genes. Silencing of p63 in MCF10A cells resulted in a 10 fold and 3.5 fold increase in the expression of DKK1 and METTL7B, respectively (Fig. 6C). These findings provide evidence that *DKK1* and *METTL7B* are direct targets of p63-mediated repression. In contrast, knockdown of p63 was associated with a decrease in the expression of *PLK2, OCEL1, TMEM205* and *TFPI2* (Fig. 6C), implicating these genes as targets for constitutive up-regulation by p63.

### Mutant p53 is co-recruited with p63 to the promoters of its target genes

**Figure 7:**
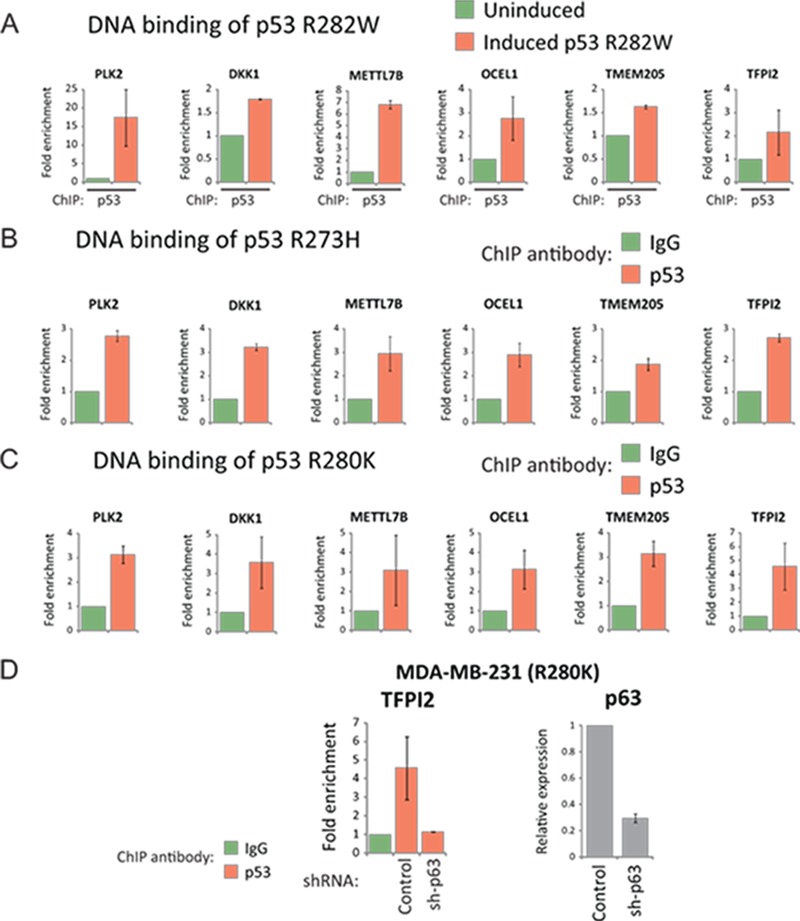
Mutant p53 associates with its target promoters through p63 (A) EI-H1299 cells with inducible p53 R282W were cultured in the presence of PonA (2.5 μg/mL) or vehicle control for 24 hours prior to ChIP analysis using a p53-specific antibody. (B) MDA-MB-468 or (C) MDA-MB-231 cells expressing the endogenous p53 R273H or R280K mutants were subjected to a ChIP analysis using either a p53-specific antibody or IgG control. (D) Polyclonal populations of MDA-MB-231 cells were generated expressing either p63-specific shRNA (sh-p63) or non-targeting shRNA (control), with reduced p63 levels confirmed by real time PCR. These cell lines were used for ChIP analysis involving the immunoprecipitation of p53 R280K as described in (B).

Our findings thus far suggest that the global targets of mutant p53 are also direct targets of p63. Furthermore, we also observed constitutive regulation of these genes by p63. Based on these results, we speculated that mutant p53 may be directly recruited to the promoters of its target genes with p63. Data from ChIP analyses were consistent with this hypothesis, as induced mutant p53 was found to be associated with these p63-REs in the promoters of *PLK2, DKK1, METTL7B, OCEL1, TMEM205* and *TFPI2* in H1299 cells (Fig. 7A). These observations were not restricted to the inducible system, as in MDA-MB-468 cells the endogenous p53 R273H mutant was also bound to these p63-REs (Fig. 7B). Further confirmation of mutant p53 recruitment to these sites was demonstrated using another endogenous p53 mutant (R280K) expressed in MDA-MB-231 (Fig. 7C). Thus, these results provide firm evidence that mutant p53 and p63 are co-recruited to these p63-REs. Silencing of p63 in MDA-MB-231 cells resulted in complete dissociation of the endogenous p53 mutant from the promoter of *TFPI2*, suggesting that mutant p53 uses p63 as a molecular chaperone to tether to these promoter regions (Fig. 7D). Collectively, these data support a model where a small subset of wild-type p53 transactivated targets are also the targets that drive mutant p53 gain-of-function. Transactivation by mutant p53 is achieved by the recruitment of p63 as a molecular chaperone that enables mutant p53 to bind to the promoters of these target genes.

### Mutant p53 induces a pro-invasive secretome

**Figure 8:**
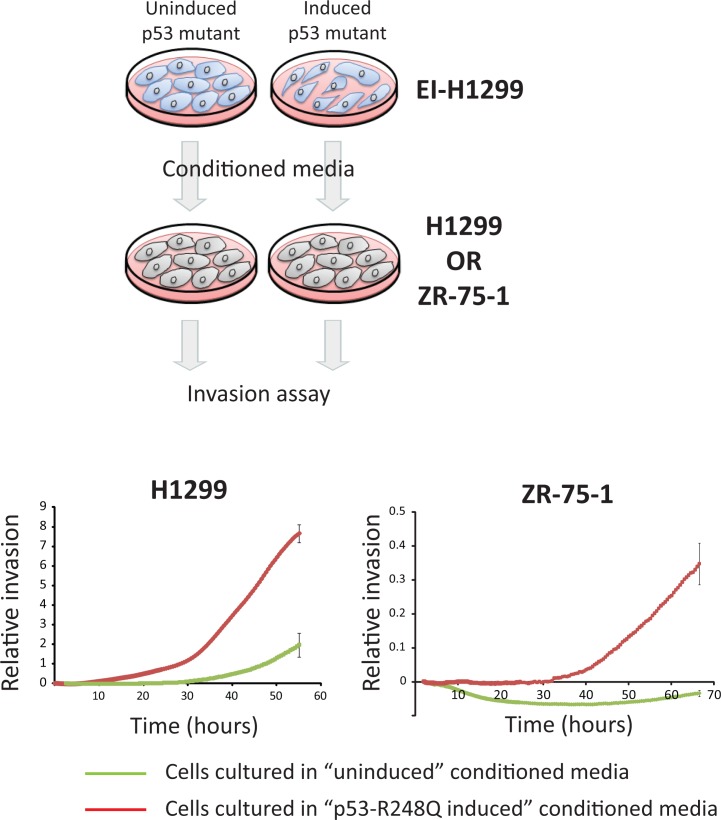
Mutant p53 induces a pro-invasive secretome EI-H1299 cells with inducible expression of the p53 R248Q mutant were cultured in the presence of PonA (2.5 μg/mL) or vehicle control for 96 hours. Independent cultures of H1299 or ZR-75-1 were grown in a dilution (50:50) of this conditioned media for 96 hours (supplemented to 10% FCS) and their invasive potential subsequently assessed in real-time using xCelligence (Roche).

An assessment of the predicted cellular localization of the 59 identified mutant p53 target genes revealed a remarkable enrichment of secreted (31%) or transmembrane (29%) proteins (Table S2). Thus, we speculated that the oncogenic transcriptional activities of mutant p53 are manifested through aberrant control of the cancer cell secretome. Using the inducible mutant p53 cell lines, we examined if the expression of p53 mutants could drive the release of pro-invasive factors. Conditioned medium was collected from either un-induced or induced p53 R248Q mutant cells following 96 hours of induction. These conditioned media were separately added in a 50:50 dilution to the primary ZR-75-1 breast epithelial cancer cells and incubated for an additional 96 hours. The capacity of these conditioned ZR-75-1 cells to invade through matrigel was subsequently assessed. ZR-75-1 cells cultured in the presence of ‘un-induced’ conditioned medium lacked the ability to invade, consistent with the epithelial characteristics of this cell line [27]. However, exposure of ZR-75-1 cells to conditioned medium from H1299 cells with induced expression of the p53 R248Q mutant enabled the cells to acquire the capacity to invade through matrigel (Fig. 8, right panel). These results suggest that the expression of mutant p53 can induce the secretion of pro-invasive factors into the surrounding microenvironment. Importantly, the pro-invasive secretome induced by the p53 R248Q mutant was not restricted to driving invasion of only the ZR-75-1 cells, as similar conditioned media also drove invasion of the parental unmodified H1299 cells (Fig. 8, left panel). Lastly, these findings were confirmed using a different p53 mutant, as conditioned media produced following induction of the p53 R175H mutant in H1299 cells could also drive the release of a pro-invasive secretome (Fig. S4). These observations implicate a role for mutant p53 in the induction of a pro-invasive cancer cell secretome.

## DISCUSSION

The broad range of cancer-associated p53 mutations and the variant genetic background of commonly used cancer cell lines have largely hampered the ability to gain a complete understanding of the global oncogenic activities of mutant p53. We have overcome this challenge through the generation of a panel of isogenic H1299 derivatives with the inducible expression of six common p53 hot spot mutants. This inducible expression system provides a sensitive platform to capture the direct transcriptional events driven by mutant p53. The expression profiling of this comprehensive panel of cell lines demonstrated that all p53 mutants share a common core set of 59 target genes. Surprisingly, the transcriptional reprogramming by mutant p53 was not related to the variant biophysical properties of the mutated protein. Such findings question the relevance of the ‘DNA contact’ or ‘structural’ classification of p53 mutants in relation to their oncogenic activities. Rather they are consistent with recent work demonstrating the critical role of the conserved aggregation signal in p53 contributing to the activity of mutant p53 proteins [28]. Furthermore, the transcriptional regulation of these 59 genes cannot be considered as a feature specific to mutant p53, as these genes were also demonstrated to represent a small (3%) proportion of the direct transcriptional activities of wild-type p53 in the H1299 inducible system. Thus it is tempting to speculate that these 59 genes represent the oncogenic ‘dark side’ of p53. This notion is supported by a previous report that one of these genes, *PLK2*, is an oncogenic direct target of wild-type p53 [29].

This study is the first to demonstrate that the gain-of-function of mutant p53 during tumorigenesis involves a collaborative approach with the p63 transcription factor to aberrantly reprogram the cancer cell transcriptome. Over half of the 59 mutant p53 target genes contained at least one putative p63 response element (Table S1C), suggesting that p63 is a critical molecular chaperone for mutant p53. Existing evidence suggests that mutant p53 also aberrantly modulates target gene expression by interacting with the NF-Y and VDR transcription factors [30, 31]. Thus, findings from this study implicate p63 as another key transcription factor utilized by mutant p53 to drive its target gene expression.

Recent insights into the global binding sites of p63 revealed that this transcription factor controls a remarkably complex downstream transcriptional network. A genome-wide tiling array identified approximately 5,800 promoters as direct putative targets of p63 [32]. The prolific DNA binding properties of the p63 transcription factor were further highlighted through a recent ChIP-seq approach which revealed 11,369 binding sites throughout the genome, with 94% of these sites containing a consensus p63-RE [26]. If mutant p53 was associated with p63 at each of its target promoters across the entire genome, then one would expect mutant p53 to drive the aberrant expression of a plethora of genes. However, our expression profiling of six different inducible mutant p53 cell lines and *in silico* analysis revealed a restricted set of genes that are regulated by both mutant p53 and p63, indicating that the co-recruitment of p63 and mutant p53 might require stringent tertiary binding structure. Alternatively, additional transcription factors might present within the complex to assist with the adaptation of a protein scaffold capable of recruiting mutant p53 and p63 onto the target gene promoter.

A widely accepted gain-of-function mechanism of mutant p53 involves its ability to sequester the TAp63 isoform from its canonical DNA response element and thereby disrupt its downstream anti-metastatic transcriptional networks [5, 19]. However, it is unclear if the ΔNp63 isoform shares a similar fate in the presence of mutant p53. Our understanding of the regulation of ΔNp63 by mutant p53 is largely limited by the absence of an antibody that can specifically detect endogenous ΔNp63. Previous studies have circumvented this limitation through the use of cell lines that highly express ΔNp63. The p63 ChIP analyses within this study were performed using the MCF-10A breast epithelial line which expresses abundant levels of the ΔNp63 isoform, thus it is tempting to speculate that ΔNp63 is the molecular chaperone used by mutant p53 to tether to its target promoters. Nevertheless, a role for the TAp63 isoform in this model cannot be excluded, as our quantitative expression analysis of p63 isoforms suggests that H1299, MB-468 and MB-231 cells almost exclusively express the TAp63 isoform (data not shown).

Our expression profiling analysis has revealed that MAP2K3 is amongst the mutant p53 transcriptional landscape. This kinase possesses biological functions consistent with a role in oncogenesis, as up-regulation of MAP2K3 is associated with the invasion and progression of breast tumors and gliomas [33]. Although we demonstrated that MAP2K3 is a direct target for mutant p53 transactivation (Fig. 2), *in silico* analysis of the *MAP2K3* promoter region did not detect a consensus p63 response element, suggesting that mutant p53 may regulate this gene through a p63-independent mechanism. Indeed, mutant p53 was recently shown to associate with the *MAP2K3* promoter through the use of NF-Y as a molecular chaperone [34]. Ectopic expression of MAP2K3 was able to rescue the proliferative defect associated with knockdown of an endogenous p53 mutant, thus demonstrating that MAP2K3 transactivation contributes significantly to the oncogenic functions of mutant p53 [34].

The downstream effectors of the 59 wild-type and mutant p53 target genes were highly enriched with genes that encode secreted protein products (Table S2). We have directly demonstrated that mutant p53 induces a pro-invasive secretomes, thus providing insight into the mechanism underlying the widely established role for mutant p53 in cancer cell invasion [5, 6, 9, 15]. Such observations are reminiscent of the hyper-secretory phenotype released from senescent cells referred to as the senescence-associated secretory phenotype (SASP) [35]. The SASP contains a plethora of biologically active molecules that collectively remodel the local and systemic tissue microenvironment [36]. Surprisingly, wild-type p53 was shown to suppress the SASP [36]. This observation is conflicting with the widely accepted role of p53 in the induction of cellular senescence, albeit in some cell types. Although we are yet to understand the complex role of p53 in the regulation of senescence and the SASP, recent evidence suggests that the activity of the mTOR pathway is the major factor influencing the senescent outcome upon p53 induction [37-40]. It is evident from this study that mutant p53 has not retained these traits of its wild-type counterpart, as it induced a pro-invasive secretome in this cell-based system. As such, our findings highlighting the divergent nature between wild-type and mutant p53 in the regulation of the secretome, thus identifying exciting new avenues to therapeutically target mutant p53-expressing tumors.

## MATERIALS AND METHODS

### Cell lines and inducible expression system

MCF10A, MDA-MB-231 and MDA-MB-468 cell lines were purchased from American Type Culture Collection (ATCC) and cultured in the recommended media supplemented with 10% FCS. The generation of ponasterone A (PonA) inducible H1299 derivatives has been previously described [15, 41]. Using p53 expression constructs encoding either wild-type p53 or the R175H, R248Q, R248W, R249S, R273H or R282W mutants (kind gifts from Dr Chikashi Ishioka, Dr Sumitra Deb and Dr Maria Lung), a panel of ecdysone-inducible H1299 cell lines were generated. Cell lines with silenced expression of p53 or p63 were generated using a pGIPZ lentiviral shRNAmir system (Open BioSystems). Briefly, HEK-293T cells were seeded at 50% confluence in a 6 well- format and transfected using the indicated pGIPZ lentiviral shRNAmir construct and the translentiviral pGIPZ packaging system (Open BioSystems) following the manusfacturer's protocol. Following 48 hours, growth medium containing viral particles was filtered and added to recipient cells seeded at 50% confluence for a further 48 hours. Growth medium was subsequently changed and cells were selected in puromycin. Polyclonal populations of selected cells were used for the necessary experiments.

### Western blot analysis and antibodies

Western blot analysis was performed as previously described [42]. Antibodies used were: mouse anti-p53 DO-1 (Santa Cruz Biotechnology, Santa Cruz, CA), mouse anti-β-actin (Sigma Aldrich), mouse anti-p21 (Thermo Scientific), mouse anti-MDM2 (clone SMP14; Santa Cruz), mouse anti-p63 H-129 (Santa Cruz) or anti-mouse IgG HRP-conjugated (GE Healthcare).

### Expression microarray analysis

The H1299 p53-WT, R175H, R248Q, R248W, R249S, R273H and R282W inducible cell lines were treated in the presence of 2.5 μg/mL PonA (or vehicle control) for 24 hours. Cells were collected and total RNA extracted using RNeasy mini kit (Qiagen) according to the manufacturer's protocol. Expression profiling was performed using Affymetrix Human Gene 1.0 ST array as per manufacturer's protocol. Two independent biological replicates of either PonA induced or vehicle control treated cultures were performed per cell line.

### Real time PCR analysis

The mRNA expression levels of specific genes of interest were determined by real time RT-PCR analysis using specific forward and reverse primers. Briefly, total RNA was extracted from cells using the RNeasy mini kit (Qiagen) according to the manufacturer's protocol. cDNA was synthesized from 1 μg RNA using random primers (Promega) and RNase H- reverse transcriptase (Promega) as per manufacturer's protocol. Primers used for specified genes are listed in Supplementary Table 3 (Table S3). Real-time PCR reactions were performed on a BioRad iCycler (BioRad) using IQ SYBR Green Supermix (BioRad) as previously described [43]. Relative target mRNA expression of specific genes was subsequently determined by the ΔΔCT method, with the levels of gene expression normalised to the average Ct value of the peptidylpolyl isomerise G (PPIG) housekeeping gene.

### Chromatin immunoprecipitation (ChIP)

The indicated H1299 p53 inducible cell lines were treated with or without 2.5μg/mL PonA for 24 hours to induce wild-type or mutant p53 expression. Cells were collected and DNA and proteins were cross-linked by addition of 1% formaldehyde for 9 min with rotation at RT. Cold glycine (625mM final concentration) was added to stop cross-linking, mixed and centrifuged for 5 minutes at 300*g*. Cells were subsequently washed twice with 50 mL cold PBS. Cell pellets were lysed in 400 μL SDS Lysis buffer (1% SDS, 10mM EDTA, 50mM Tris-HCl pH 8.1) with protease inhibitors, followed by sonication (6 × 15 sec; 30% amplitude, 3mm tip, Sonics Vibra Cell sonicator). Following clarification, lysates were diluted 10-fold in dilution buffer (0.01% SDS, 1.1% Triton X-100, 1.2mM EDTA, 16.7mM Tris-HCl pH 8.1, 167mM NaCl) and inputs taken. Lysates were precleared with Protein A sepharose beads with BSA and sonicated salmon sperm DNA (ssDNA) at 4°C with rotation for 2 hours. Lysates were subsequently incubated with 4 μg of anti-p53, anti-p63 or mouse IgG at 4°C with rotation overnight. Immune complexes were precipitated with Protein A sepharose with ssDNA at 4°C with rotation for 2 hours. Beads were washed once each with low salt immune complex wash buffer (20mM Tris-HCl pH 8, 150mM NaCl, 2mM EDTA, 1% Triton X-100, 0.1% SDS), high salt immune complex wash buffer (20mM Tris-HCl pH 8, 500mM NaCl, 2mM EDTA, 1% Triton X-100, 0.1% SDS), LiCl immune complex wash buffer (10mM Tris-HCl pH 8, 1mM EDTA, 0.25M LiCl, 1% NP-40, 1% sodium deoxycholate) and twice with TE buffer (10mM Tris-HCl pH 8, 1mM EDTA). Specific immune complexes were eluted in 250μL SDS Elution Buffer (1% SDS, 0.1M NaHCO_3_). Cross-links were reversed by addition of 10μL 5M NaCl and heating at 65°C for 16 hours, followed by addition of 10μL 0.5M EDTA, 20μL 1M Tris-HCl pH 6.5 and 4μL 10mg/mL Proteinase K and heating at 45°C for 1 hour. DNA was purified using a PCR Purification kit following the manufacturers protocol (Qiagen). Levels of gene specific promoter DNAs were determined by real-time PCR using primers spanning the p53 response elements (Table S3). Relative binding was normalised against two independent negative control regions of non-related genomic DNA (adjacent to *β-GLOBIN* and *CDC25B* genes). ChIP data is presented as the mean ± SE of between two and four independent biological replicates.

### Cell proliferation assays

Proliferation assays were performed in real-time through collection of phase contrast images at 30 minute intervals using Incucyte (Essen). The indicated H1299 p53 inducible cells were seeded at ~10% confluence, induced with PonA (2.5μg/mL) and their proliferation monitored through the acquisition of phase contrast images at 15 minute intervals and analysed using Incucyte software (Essen).

### Cell cycle analysis

Both adherent and detached cells in the growth media were harvested, washed twice with cold PBS, fixed in ice-cold 70% ethanol and incubated overnight at 4°C. Cells were stained with 50 μg/mL propidium iodide solution (Sigma Aldrich) and 100 μg/mL RNase A (Sigma Aldrich) for 45 minutes at 37°C. DNA content was determined with the use of a FACSCalibur™ flow cytometer (BD, CA, USA) with cell cycle profiles analyzed using WinMDI v2.8 software (Scripps Research Institute).

### In silico analysis of p53 and p63 response elements

p53scan or p63scan [44] were used to identify putative p53-REs or p63-REs. Gene sequences were derived from NCBI, with Aceview used to define the classical promoter region (10kB upstream from the initiation site), 1^st^ intron or 3'UTR.

## Supplementary Figures and Tables

Supplementary Figures

Supplementary Tables
