# Supplementary material for: Mutant p53 uses p63 as a molecular chaperone to alter gene expression and induce a pro-invasive secretome
Source: Oncotarget. 2011 Dec 25;2(12):1203–17. doi: 10.18632/oncotarget.382 (PMC3282078; doi:10.18632/oncotarget.382)
Supplement: Supplementary Figures [file oncotarget-02-1203-s001.pdf]

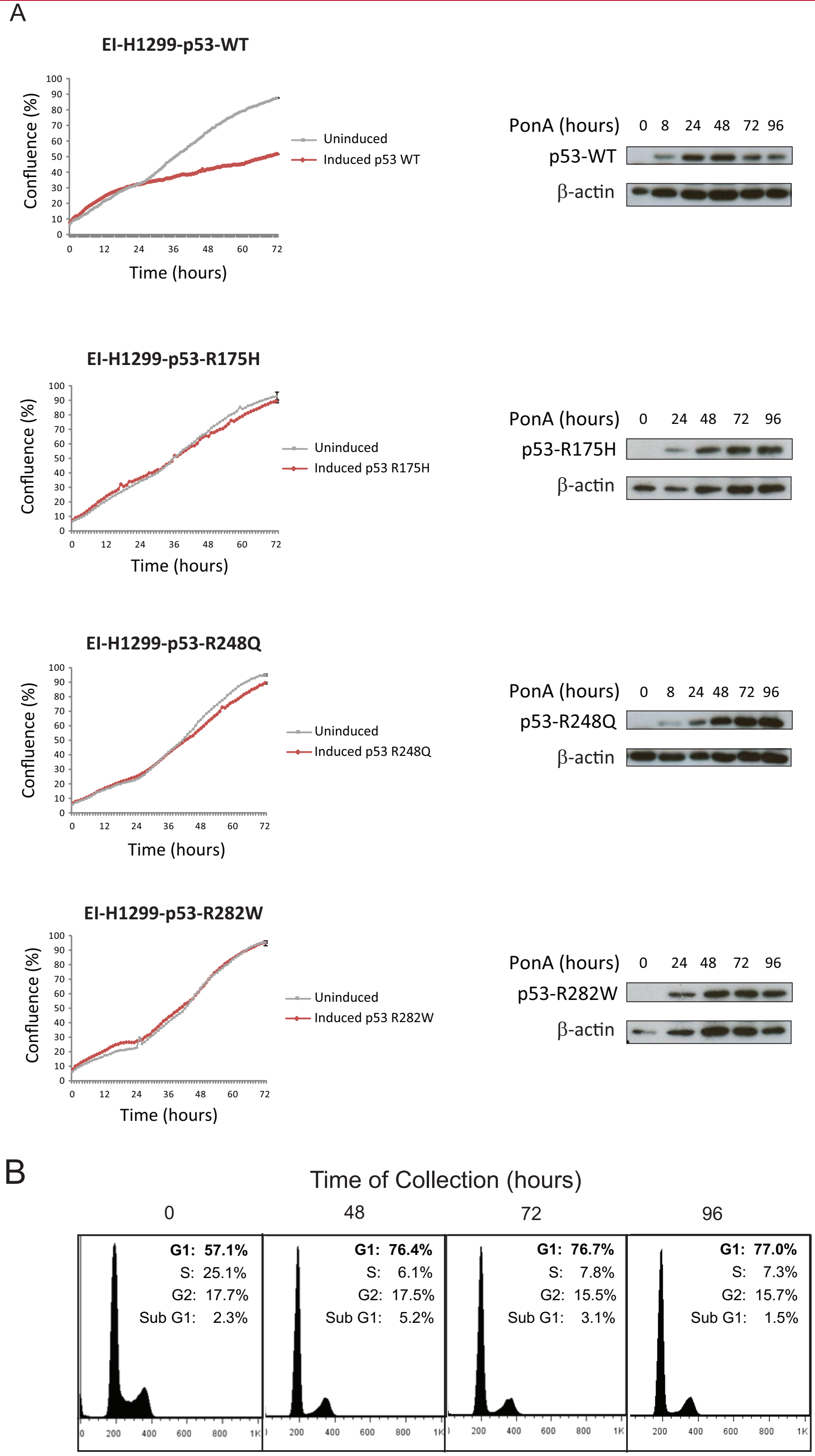

Supplementary Figure S1: Biological outcome of p53 induction. (A) EI-H1299 cells with inducible p53-WT, R175H, R248Q or R282W were cultured in PonA (2.5 µg/mL) or vehicle control over a 72 hour period and their proliferation assessed using Incucyte (Essen). Kinetics of p53 protein induction during this timecourse was determined using Western blot analysis with β-actin used as a loading control. (B) The EI-H1299 cells with inducible wild-type p53 were induced with 2.5 µg/mL PonA for 0, 48, 72 or 96 hours. Cell cycle analysis was performed by propidium iodide staining.

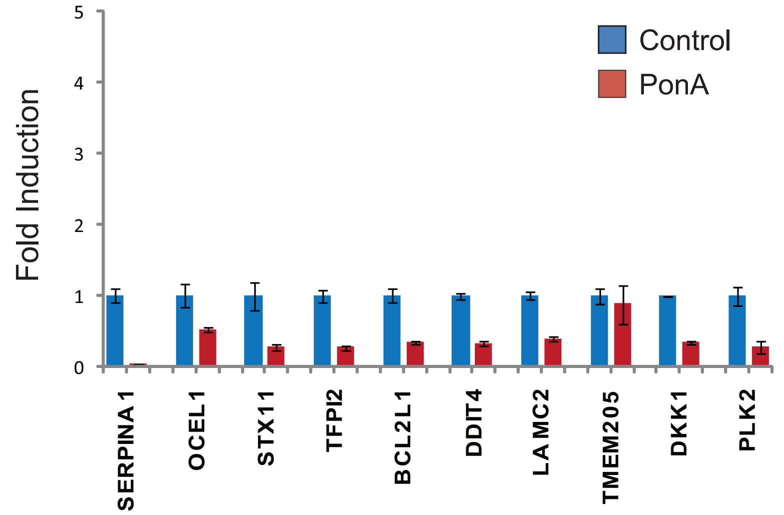

Supplementary Figure S2: The inducing agent (PonA) does not up-regulate mutant p53 targets in the absence of mutant p53. The expression of the ten validated mutant p53 targets (from Figure 2) was determined following exposure of PonA (2.5µg/mL) or vehicle control (Control) to the parental H1299 inducible line (p53 null) for 24 hours.

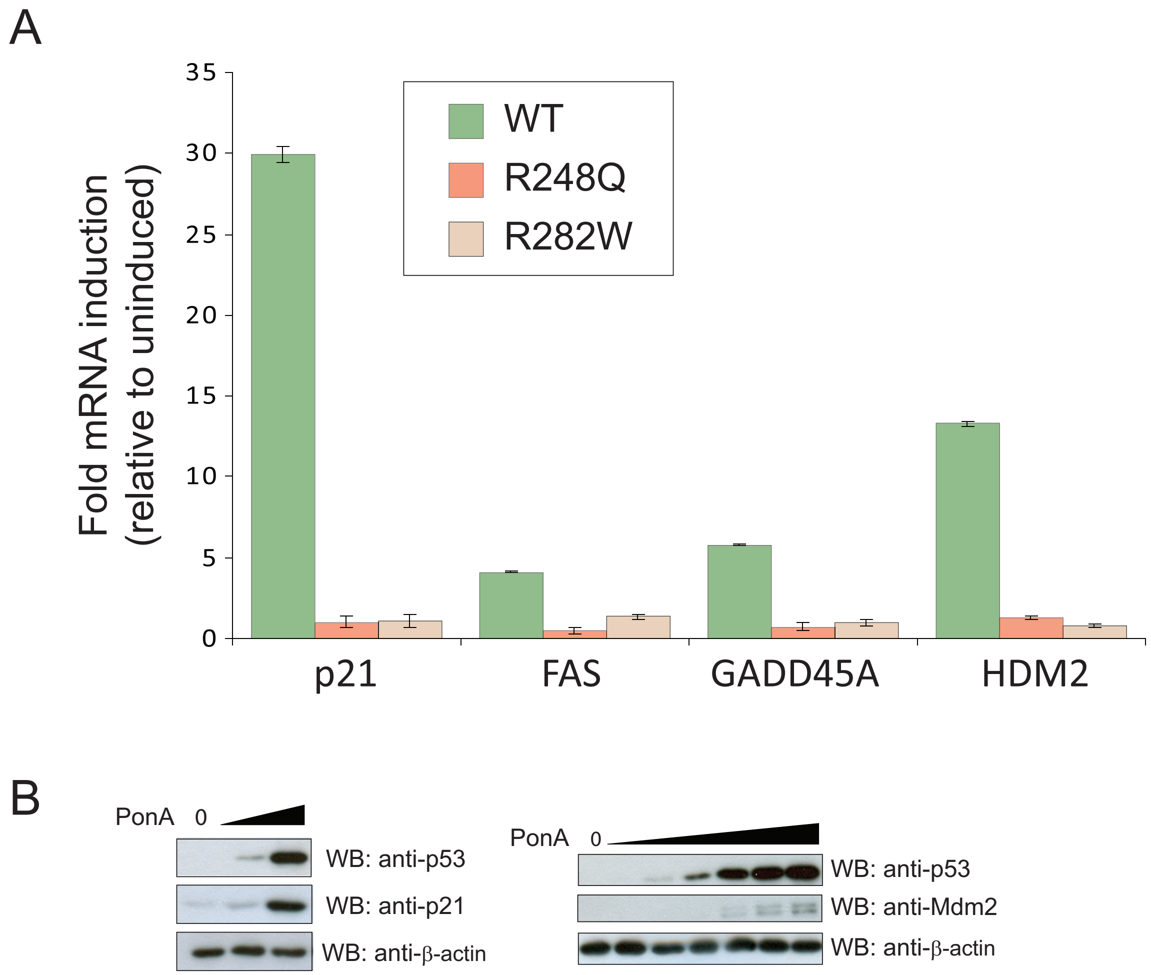

Supplementary Figure S3: Characterisation of transcriptional activity of inducible wild-type and mutant p53 H1299 cells. (A) The expression of four common p53 target genes (*p21*, *FAS*, *GADD45A* and *MDM2*) was determined following 24 hour induction of p53 (WT, R248Q or R282W) using PonA (2.5 µg/mL). Data is presented as fold change from each uninduced control (uninduced = 1). (B) Western blot analysis of p21 and MDM2 protein levels in EI-H1299-p53-WT cells following titration of PonA for 24 hours. β-actin was used as a loading control.

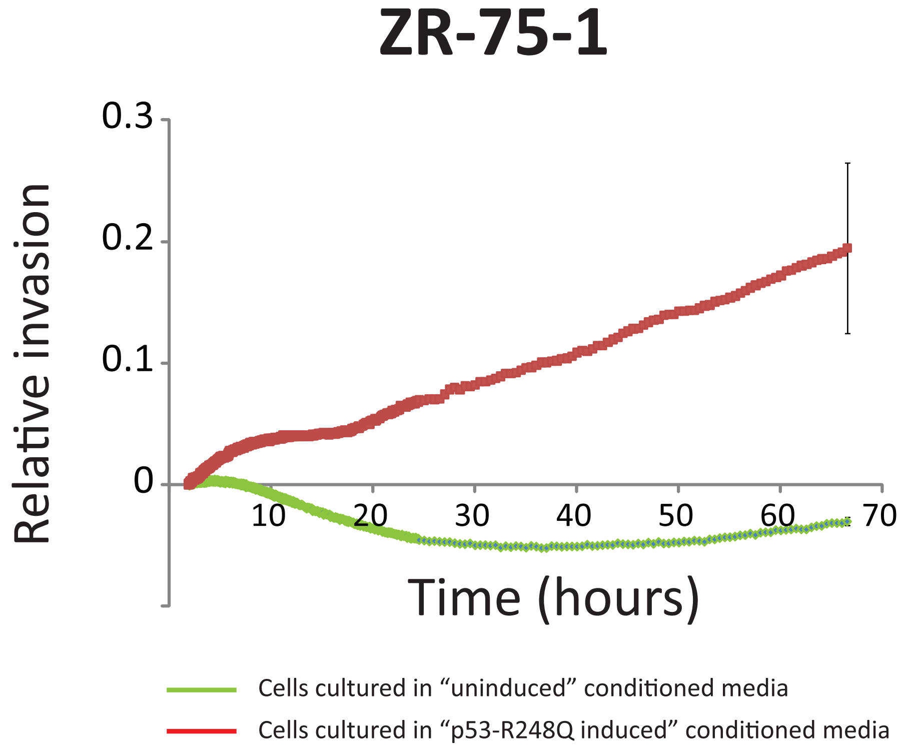

Supplementary Figure S4: The p53 R175H mutant induces a pro-invasive secretome. EI-H1299 cells with inducible expression of the p53 R175H mutant were cultured in the presence of PonA (2.5 μg/mL) or vehicle control for 96 hours. Independent cultures of ZR-75-1 were grown in a dilution (50:50) of this conditioned media for 96 hours and their invasive potential subsequently assessed in real-time using xCelligence (Roche).
