# Supplementary material for: Mutant p53 uses p63 as a molecular chaperone to alter gene expression and induce a pro-invasive secretome
Source: Oncotarget. 2011 Dec 25;2(12):1203–17. doi: 10.18632/oncotarget.382 (PMC3282078; doi:10.18632/oncotarget.382)
Supplement: Supplementary Tables [file oncotarget-02-1203-s002.pdf]

Supplementary Table 1.

(A) p53Scan results of promoters of mutant p53 target genes

Gene	Start	End	p53 scan score	Sequence	Spacer	Sequence	Location
ACAD11	32992	33024	14.6	AGGCAGGCCC	CCAGTCCATTGT	GGGCATGTCC	no site
AHCYL1	4732	4752	6.2	TGTGTTGCCC		AGGCTTGTCT	1st intron
AKAP12							no site
AKAP13	29610	29630	9.1	AAGCAAGTCT		GGTCATACCT	1st intron
AKAP13	103471	103491	10.5	AGGCAAGTCT		GAGCAGGCCC	1st intron
ANGPTL4	-4133	-4113	6.9	AGACCAGCCT		GGACATGGCG	Promoter
ATXN1							no site
BCL2L1							no site
BHLHE41	-8624	-8604	9.0	GGACAAGCTC		CTGCAAGTCA	Promoter
INO80C	13432	13452	7.6	AAAAAAGTTT		GTACATGTTT	1st intron
CCDC80							no site
CTH							no site
DDIT4	-3155	-3135	14.9	AAGCATGTCT		AGGCAAGCCC	Promoter
DENND1A							no site
DKK1	-7008	-6988	5.5	TAACATGAAA		ATACTAGCTT	Promoter
EDNRA							no site
EHHADH							no site
FAM125B							no site
FSTL3	5327	5347	5.5	GGACAGGCCT		GGGCCTGGTG	3' UTR
GP6							no site
GPR17	-7623	-7603	5.3	GGACCTGGCA		GGGCTAGTCT	Promoter
HSPA5							no site
ITGA3	-2213	-2193	5.8	GCTTTTGTTT		AGGCATGTCT	Promoter
KLHL5							no site
LAMC2							no site
LAPTM5	9538	9558	13.8	AGACAAGCCC		GGGCCTGTCT	1st intron
LRIG1	13707	13727	9.9	AGACATGTCC		ATACATGGAA	1st intron
CCDC159	-4579	-4559	5.1	AAACAAGACA		AAACAGGCTG	Promoter
MAP2K3							no site
METTL7B	-4993	-4973	8.3	ATACATGCTA		CAACATGTGT	Promoter
NR2F2	-9706	-9686	5.6	CAACATGCTC		TGGCTGGCTG	Promoter
OCEL1	-6934	-6914	5.0	AGAGATGCTA		GGACTGGCCC	Promoter
P2RX4	6816	6836	7.6	AGGCGTGTTG		GCGCATGCCT	1st intron
PDE2A	30573	30593	13.7	GAGCATGCCA		GAGCATGCCT	1st intron
PLCL1	57687	57707	9.7	GTACATGCTA		CTACTTGTCT	1st intron
PLCL1	112187	112207	12.9	AGACAAGATT		AGGCATGTTC	1st intron
PLCL1	195614	195634	7.4	ATACATGTAC		AGGTTTGTTA	1st intron
PLCXD2							no site
PLEKHA2	-8602	-8582	10.2	CAGCATGCCC		AGACATGGCA	Promoter
PLK2	-2207	-2187	16.4	AAACATGCCT		GGACTTGCCC	Promoter
PRSS12							no site
PSD3	61338	61358	9.6	TGGCAAGCTC		AAACAAGCAT	1st Intron
PSD3	484223	484243	14.1	TGACATGTCT	GGGACCTC	TGACATGTGT	3' UTR
PTGIR	-6020	-6000	6.8	GGGCAGGTCC		CTGCGTGCCC	Promoter
RDH5	455	475	6.1	AGGCAAGCAA		GAAGATGCCT	1st intron

SERPINA1							no site
SEMA3C							no site
SETX							no site
SFRP1							no site
SLC35F5	42127	42147	7.4	AAGCATGTTT		GTGCAAATTT	3' UTR
SNTB1							no site
SRR	10656	10676	7.2	GGGCATGGTG		GTGCATGCCT	1st intron
STC1							no site
STX11	-9986	-9966	6.7	AGGCATGAAC		CATCATGCCT	Promoter
TFPI2	-9250	-9230	5.8	GGACAAGTGC		TGAGATGCTA	Promoter
TGFBI							no site
TMCC3	64742	64770	14.0	AGACAAGCAC	AGAGCAAC	AGGCATGTCA	1st intron
TNFSF4	-9836	-9816	11.2	AGACAAGCAC		ATACATGCAT	Promoter
TCEAL1							no site
TMEM205							no site
WBP5	-5921	-5901	9.4	ATGCAGGTTT		GTGCATGTTT	Promoter
CD22							no site
CDO1	-1850	-1830	5.4	TTGCAAATTT		AAACATGCAC	Promoter

(B) p53Scan results of promoters of genes unique to wild-type p53

Gene	Start	End	p53 scan score	Sequence	Spacer	Sequence	Location
ABCA1							No site
ABHD4							No site
ACTA2							No site
ANKRA2	-9543	-9523	10.1	TAACATGTGT		AAACTTGCCA	Promoter
APLP1							No site
AREG	-4849	-4829	11.5	TGGCTTGCCA		AAACATGTTA	Promoter
ASAH3L							No site
BTG2	-1894	-1874	11.8	ACGCATGTTC		AGGCATGCAT	Promoter
C10orf141							No site
C12orf5							No site
C6orf138							No site
C9orf19	-7721	-7691	13.8	TGACATGTTT	GCTTTAAGCG	TAACATGTTT	Promoter
CDKN1A	-2233	-2213	13.0	GAACATGTCC		CAACATGTTG	Promoter
CES2	-6350	-6330	14.2	GGGCATGTTC		ATACATGTCA	Promoter
CLCA2							No site
CMBL							No site
CSF1							No site
CYFIP2							No site
DCLK1							No site
DDB2							No site
DGKA	-6793	-6773	6.2	GTGCATGCTC		ACACCTGTAA	Promoter
DQX1	-138	-118	10.4	CTACAAGTCC		CAGCATGCCT	Promoter
EBI3							No site
EDIL3							No site
EPS8L2							No site
FAM13C1							No site
FAS							No site
FDXR							No site
GAL3ST4	-4417	-4397	5.4	GGGCATGATG		GCTCATGCCT	Promoter
GDF15	-850	-830	10.7	CATCTTGCCC		AGACTTGTCT	Promoter
GLS2							No site
GPC1							No site
GPR87							No site
GRHL3							No site
INPP5D							No site
LRP1	-687	-667	10.4	GGACAAGCTC		CGGCGTGTCC	Promoter
MAST4							No site
ORAI3	-8603	-8583	12.3	AGGCATTTCT		GGACATGCCT	Promoter
PLEKHG1							No site
PLK3	-440	-420	5.3	TAACATGCCC		GGGCAAAAGC	Promoter
PLXNB3	-2388	-2368	8.6	GTGTAAGCCA		AGACATGCAC	Promoter

PSTPIP2							No site
RGL1							No site
RPS27L							No site
RRAD	-2577	-2557	14.2	TGACATGTAT		GAACATGCCC	Promoter
RRM2B							No site
SCN2A							No site
SESN1							No site
SESN2							No site
SFRP4							No site
SLC44A5							No site
TMEM16D							No site
TP53I3	-1942	-1922	9.8	ATACATCCTC		AGACATGTTT	Promoter
TP53INP1							No site
TRIM22							No site
VCAN							No site
WDR63							No site
ZMAT3							No site
ZNF385A	-7701	-7681	8.3	GGGCATGATG		GCACATGCCT	Promoter

(C) p63Scan results from promoters of mutant p53 target genes

Gene	Start	End	p63 scan score	Sequence	Sequence	Location
ACAD11						no site
AHCYL1						no site
AKAP12						no site
AKAP13	1398	1417	8.6	TACAAGTTT	TGTCAAGTTT	1st intron
ANGPTL4	-4133	-4113	8.3	AGACCAGCCT	GGACATGGCG	Promoter
ATXN1						no site
BCL2L1						no site
BHLHE41	-8624	-8604	9.0	GGACAAGCTC	CTGCAAGTCA	Promoter
INO80C	10286	10305	8.7	AACATGTGA	GGACATGAGA	1st intron
CCDC159						no site
CCDC80						no site
CTH	-5236	-5217	9.1	GACCAGCCT	CGGCCTGCCA	Promoter
DDIT4	-3155	-3135	11.9	AAGCATGTCT	AGGCAAGCCC	Promoter
DENND1A	6012	6031	9.5	TCCATGTTG	GAGCATGCAT	1st intron
DKK1	-7008	-6988	7.2	TAACATGAAA	ATACTAGCTT	Promoter
EDNRA						no site
EHHADH						no site
FAM125B						no site
FSTL3	-9353	-9334	7.7	GACAGGTGT	GGGCTTGGTT	Promoter
GP6						no site
GPR17	-7623	-7603	9.0	GGACCTGGCA	GGGCTAGTCT	Promoter
HSPA5	-1716	-1697	6.1	GGCATGCAC	CACCACGCCC	Promoter
ITGA3						no site
KLHL5						no site
LAMC2						no site
LAPTM5	9538	9558	12.0	AGACAAGCCC	GGGCCTGTCT	1st intron
LRIG1						no site
MAP2K3						no site
METTL7B	-4993	-4973	10.4	ATACATGCTA	CAACATGTGT	Promoter
NR2F2	-9706	-9686	9.1	CAACATGCTC	TGGCTGGCTG	Promoter
OCEL1	-3187	-3168	6.3	GGCATGGTG	GCTCATGTCT	Promoter
P2RX4	6816	6836	9.6	AGGCGTGTTG	GCGCATGCCT	1st intron
PDE2A	30573	30593	12.6	GAGCATGCCA	GAGCATGCCT	1st intron
PLCL1	57687	57707	10.5	GTACATGCTA	CTACTTGTCT	1st intron
PLCXD2						no site
PLEKHA2	-8602	-8582	10.7	CAGCATGCCC	AGACATGGCA	Promoter
PLK2	-2207	-2187	13.1	AAACATGCCT	GGACTTGCCC	Promoter
PRSS12	5800	5819	9.5	TGCATGCAC	ACGCATGTGT	1st Intron
PSD3	61338	61358	10.6	TGGCAAGCTC	AAACAAGCAT	1st Intron
PTGIR	-1240	-1221	8.8	TGCAGGCCG	AGGCTGGCCA	Promoter
RDH5						no site

SERPINA1						no site
SEMA3C						no site
SETX	-4004	-3985	10.8	CACAAGTTC	TGACTTGTCT	Promoter
SFRP1						no site
SLC35F5						no site
SNTB1						no site
SRR	10656	10676	8.2	GGGCATGGTG	GTGCATGCCT	1st intron
STC1						no site
STX11	-9986	-9966	9.5	AGGCATGAAC	CATCATGCCT	Promoter
TFPI2	-7021	-7002	6.7	GTCAAGTTT	GCTCTTGACA	Promoter
TGFBI						no site
TMCC3						no site
TNFSF4	-9836	-9816	11.1	AGACAAGCAC	ATACATGCAT	Promoter
TCEAL1	-3848	-3829	6.6	GGCTGGACA	GGGCCTGCTG	Promoter
TMEM205	-2538	-2519	8.3	AGCAGGCAA	ACACATGCTG	Promoter
WBP5	-5921	-5901	10.7	ATGCAGGTTT	GTGCATGTTT	Promoter
CD22						no site
CDO1	-4848	-4829	8.2	ACCATGTCA	ATGCCTTGTCT	Promoter

Supplementary Table 2. Predicted cellular localization of mutant p53 targets (derived from NCBI gene entry or *Genecard*).

Accession Number	Gene Symbol	Gene Name	Cellular Localization	Secretome Reference
NM_002560	**P2RX4**	Purinergic receptor P2X, ligand-gated ion channel, 4	Plasma membrane	
NM_006528	**TFPI2**	Tissue factor pathway inhibitor 2	**Secreted**	(1)
NM_001002236	**SERPINA1**	Serpin peptidase inhibitor, clade A	**Secreted**	(1)
NM_002905	**RDH5**	Retinol dehydrogenase 5	**Secreted**	(1)
NM_025181	**SLC35F5**	Solute carrier family 35, member F5	Plasma membrane	
NM_199511	**CCDC80**	Coiled-coil domain containing 80	**Secreted**	(2)
NM_001902	**CTH**	Cystathionase	Cytoplasm	
NM_003764	**STX11**	Syntaxin 11	Membrane / golgi	
NM_002599	**PDE2A**	Phosphodiesterase 2A	Plasma membrane	
NM_012242	**DKK1**	Dickkopf homolog 1	**Secreted**	(1)
NM_000358	**TGFBI**	Transforming growth factor, beta-induced	**Secreted**	(1)
BC071561	**LRIG1**	Leucine-rich repeats and immunoglobulin-like domains 1	Plasma membrane	
NM_032169	**ACAD11**	Acyl-Coenzyme A dehydrogenase family, member 11	Peroxisome	
NM_021947	**SRR**	Serine racemase	Unknown	
NM_019058	**DDIT4**	DNA-damage-inducible transcript 4	Cytoplasm	
NM_000332	**ATXN1**	Ataxin 1	Cytoplasm / nucleus	
NM_006622	**PLK2**	Polo-like kinase 2	Nucleus	
BC025968	**BHLHB3**	Basic helix-loop-helix domain containing, class B, 3	Nucleus	
NM_020946	**DENND1A**	DENN/MADD domain containing 1A	Cell junction	
NM_001957	**EDNRA**	Endothelin receptor type A	Plasma membrane	
NM_002756	**MAP2K3**	Mitogen-activated protein kinase kinase 3	Cytoplasm	
NM_015310	**PSD3**	Pleckstrin and Sec7 domain containing 3	Cell junction	
NM_003012	**SFRP1**	Secreted frizzled-related protein 1	**Secreted**	(1)
NM_021021	**SNTB1**	Syntrophin, beta 1	**Secreted**	(1)
NM_005860	**FSTL3**	Follistatin-like 3	**Secreted**	(1)(3)
NM_006738	**AKAP13**	A kinase (PRKA) anchor protein 13	Cytoplasm / nucleus / plasma membrane	
NM_005562	**LAMC2**	Laminin, gamma 2	**Secreted**	(1)
NM_003155	**STC1**	Stanniocalcin 1	**Secreted**	(1)
NM_001966	**EHHADH**	Enoyl-Coenzyme A, hydratase/3-hydroxyacyl Coenzyme A dehydrogenase	Peroxisome	
NM_015046	**SETX**	Senataxin	Nucleus	
BC004121	**OCEL1**	Occludin/ELL domain containing 1	Unknown	
AY358949	**TMEM205**	Transmembrane protein 205	Plasma membrane	
NM_033446	**FAM125B**	Family with sequence similarity 125, member B	Endosome / membrane	
NM_006762	**LAPTM5**	Lysosomal associated multispanning membrane protein 5	Lysosome / membrane	
NM_004780	**TCEAL1**	Transcription elongation factor A (SII)-like 1	Nucleus	
NM_005100	**AKAP12**	A kinase (PRKA) anchor protein (gravin) 12	Cytoplasm	
NM_139314	**ANGPTL4**	Angiopoietin-like 4	**Secreted**	(1)
NM_015990	**KLHL5**	Kelch-like 5	Cytoplasm	
NM_003619	**PRSS12**	Protease, serine, 12	**Secreted**	(1)
NM_003326	**TNFSF4**	Tumor necrosis factor (ligand) superfamily, member 4	**Secreted**	(1)
NM_006621	**AHCYL1**	S-adenosylhomocysteine hydrolase-like 1	Endoplasmic reticulum	
NM_005347	**HSPA5**	Heat shock 70kDa protein 5	**Secreted**	(1)
NM_006226	**PLCL1**	Phospholipase C-like 1	**Secreted**	(1)
NM_153268	**PLCXD2**	Phosphatidylinositol-specific phospholipase C, X domain containing 2	Unknown	
NM_021623	**PLEKHA2**	Pleckstrin homology domain containing, family A	Cytoplasm / membrane	
NM_138578	**BCL2L1**	BCL2-like 1	Mitochondria	
NM_006379	**SEMA3C**	Sema domain, immunoglobulin domain (Ig), short basic domain, secreted 3C	**Secreted**	(1)
NM_016303	**WBP5**	WW domain binding protein 5	Unknown	
NM_000960	**PTGIR**	Prostaglandin I2 (prostacyclin) receptor	Plasma membrane	
NM_001083899	**GP6**	Glycoprotein VI	Plasma membrane	
NM_001080503	**CCDC159**	Coiled-coil domain containing 159	Unknown	
NM_002204	**ITGA3**	Integrin, alpha 3	Plasma membrane	
NM_001801	**CD22**	CD22 molecule	Plasma membrane	
NM_001771	**CDO1**	Cysteine dioxygenase, type I	Cytoplasm	
NM_152637	**METTL7B**	Methyltransferase like 7B	**Secreted**	(3)
NM_005291	**GPR17**	G protein-coupled receptor 17	Plasma membrane	
NM_020698	**TMCC3**	Transmembrane and coiled-coil domain family 3	Plasma membrane	
NM_021005	**NR2F2**	Nuclear receptor subfamily 2, group F, member 2	Nucleus	
NM_001098817	**INO80C**	INO80 complex subunit C	Nucleus	

Supplementary Table 3. Primer sequences

Primer Name	Purpose	Sequence (5'→3')	Direction
Beta-Globin-ChIP-F	ChIP - neg control	GAAGAGCCAAGGACAGGTAC	Forward
Beta-Globin-ChIP-R	ChIP - neg control	CAACTTCATCCACGTTCACC	Reverse
BCL2L1-F	Real-time PCR	GGGGTAAACTGGGGTCGCATT	Forward
BCL2L1-R	Real-time PCR	ACCAGCGGTTGAAGCGTTC	Reverse
CDC25B-ChIP-F	ChIP - neg control	GCTGTGGTGTGTAGGAAGCAGCAG	Forward
CDC25B-ChIP-R	ChIP - neg control	AAAGGAGGCCAAAGTCGGGCAATG	Reverse
DDIT4-F	Real-time PCR	TTTGGGACCGCTTCTCGTCG	Forward
DDIT4-R	Real-time PCR	GTAAGCCGTGTCTTCCTCCGG	Reverse
DKK1-ChIP-F	ChIP	CTTCTTTTGCCCCAGGCAATGGA	Forward
DKK1-ChIP-R	ChIP	CCGCCTGCCAATCAGAAGGTTGA	Reverse
DKK1-F	Real-time PCR	AACGCTGCATGCGTCACGCTAT	Forward
DKK1-R	Real-time PCR	TGAGGCACAGTCTGATGACCGGA	Reverse
FAS-F	Real-time PCR	ATGCTGGGCATCTGGACCCT	Forward
FAS-R	Real-time PCR	GCCATGTCCTTCATCACACAA	Reverse
GADD45A-F	Real-time PCR	GGAGGAAGTGCTCAGCAAAG	Forward
GADD45A-R	Real-time PCR	ATCTCTGTCGTCGTCCTCGT	Reverse
GPR17-F	Real-time PCR	GTCCTAGGGAAAGTGCCAGCCG	Forward
GPR17-R	Real-time PCR	GCCCACCAACTCCGTTTGGACA	Reverse
MDM2-F	Real-time PCR	TCTACAGGGACGCCATCGA	Forward
MDM2-R	Real-time PCR	CTGATCCAACCAATCACCTGAA	Reverse
INO80C-F	Real-time PCR	AGGGCATTGCCGTGGCAACT	Forward
INO80C-R	Real-time PCR	ACGACGTCAGAGGGCAGCCT	Reverse
LAMC2-F	Real-time PCR	TGAACAGGAGATTGGGAGTCTGAA	Forward
LAMC2-R	Real-time PCR	GACATCATGGGCCGCAGTTG	Reverse
METTL7B-ChIP-F	ChIP	CATATCTGTACAATGCAATGCTACTTGGAC	Forward
METTL7B-ChIP-R	ChIP	TCCCCATCCCAACCCCAGTACCA	Reverse
METTL7B-F	Real-time PCR	GAGCGGTTTGTGGTGGCTCCTG	Forward
METTL7B-R	Real-time PCR	GCCACATGAAGGCCCAGCTTCC	Reverse
NR2F2-F	Real-time PCR	TGCGGAGACAAGTCGAGCGG	Forward
NR2F2-R	Real-time PCR	GGCATCCTGCCCCTCTGCAC	Reverse
OCEL1-ChIP-F	ChIP	TGGCAGCTGAGCTAGACCTCG	Forward
OCEL1-ChIP-R	ChIP	GTCCCCACACCTGGTTGTCCTTC	Reverse
OCEL1-F	Real-time PCR	CCCCACCCAGTGCCCGACTA	Forward
OCEL1-R	Real-time PCR	GCAGCGAGCCTGCTTGTCCA	Reverse
p21-F	Real-time PCR	TGGACCTGGAGACTCTCAGGGTCG	Forward
p21-R	Real-time PCR	TTAGGGCTTCCTCTTGGAGAAGATC	Reverse
p63-F	Real-time PCR	TTCTTAGCGAGGTTGGGCTG	Forward
p63-R	Real-time PCR	GATCGCATGTCGAAATTGCTC	Reverse
PLK2-ChIP-F	ChIP	CACCCCTAAGCCCCACCCCTTA	Forward
PLK2-ChIP-R	ChIP	AGGCTTTGCCATTAGCGAGGAGAA	Reverse

PLK2-F	Real-time PCR	ACCAGCTCTCAGACCACACCGT	Forward
PLK2-R	Real-time PCR	CCAGACATCAGCAGAGTTGTCAGCC	Reverse
SERPINA1-F	Real-time PCR	GTAGGCGGGCGACTCAGATC	Forward
SERPINA1-R	Real-time PCR	GACGGCATTGTCGATTCACTGT	Reverse
STX11-F	ChIP	TCGCACTCTCGCTCCCAGTCC	Forward
STX11-R	ChIP	TGTCCTCGTGGGGCGAGTCAA	Reverse
TFPI2-ChIP-BS1-F	ChIP	AAAGGACAGTTGCTTTGGGTGTCA	Forward
TFPI2-ChIP-BS1-R	ChIP	TGGGAAGAGGAAGAACAGGAAGTGC	Reverse
TFPI2-F	Real-time PCR	TGGGCTTCTGCGCACCAAAGA	Forward
TFPI2-R	Real-time PCR	GCACGTTTGCAATCCTCCCTGC	Reverse
TMCC3-F	Real-time PCR	ACCGTGGACCGGACCTACTCG	Forward
TMCC3-R	Real-time PCR	GCCTGCTGCTTGTCTGCGTTG	Reverse
TMEM205-ChIP-F	ChIP	CAGCCCCTCCCAGTGTCCCC	Forward
TMEM205-ChIP-R	ChIP	ACAGCGACCCTCTTTTGGCCG	Reverse
TMEM205-F	Real-time PCR	CAGGTGCCTGGGGCATGCAA	Forward
TMEM205-R	Real-time PCR	GCCCACATGGCAGCTGTGGT	Reverse
